# Impact of Partially Substituting Canola Meal with Solvent-Extracted Distillers Grain with Solubles as a Protein Source on Milk Production in a Commercial Holstein Dairy Herd

**DOI:** 10.3390/ani13132192

**Published:** 2023-07-04

**Authors:** Randy J. Edwards, David N. Ledgerwood, Fernanda C. Ferreira, Heidi A. Rossow

**Affiliations:** 1 Veterinary Medicine Teaching and Research Center, University of California, Davis, 18830 Road 112, Tulare, CA 93274, USAfcferreira@ucdavis.edu (F.C.F.); 2 Novita Nutrition, Brookings, SD 57006, USA

**Keywords:** partial budget analysis, protein supplement, feeding decisions

## Abstract

**Simple Summary:**

The rising cost of canola meal has led dairy producers and nutritionists to explore alternative protein sources, such as solvent-extracted dried distillers grains with solubles (SDG). Adding SDG to a ration can potentially minimize feed costs while maximizing production efficiency; however, no research has yet compared the economic impact of feeding cows canola meal vs. SDG. The nutrient profile diversity between fincanola meal and SDG may be helpful when meeting metabolizable protein requirements for dairy cattle.

**Abstract:**

The removal of corn oil from dried distillers grains using solvent extraction allows a higher level of inclusion for solvent-extracted dried distillers grains with solubles (SDG) in rations and reduces the risk of milk fat depression in lactating Holstein dairy cows. The objective of this study was to evaluate the impact of substituting 70% of the canola meal (CM) with SDG on milk production and total mixed ration costs. A total of 1408 Holstein cows averaging 91 ± 49 days in milk were randomly enrolled to one of four dietary treatment groups: (1) primiparous control cows (PC) fed 13% CM and 0.21% urea; (2) primiparous SDG cows (PSDG) fed 4.2% CM, 8.8% SDG and 0.42% urea; (3) multiparous control cows (MC) with 13% CM and 0.21% urea; and (4) multiparous SDG cows (MSDG) with 4.1% CM, 8.6% SDG and 0.42% urea. The total mixed rations were formulated to be isonitrogenous. For the income over the feed costs from a control herd, the fed PC and MC’s total mixed rations and the fed PSDG and MSDG’s total mixed rations were compared. The milk yield, energy-corrected milk, milk fat yield, milk protein yield and milk protein % were lower in the PC cows compared to the PSDG cows. The MSDG cows scored lower in terms of the milk yield, milk protein yield and milk protein % and higher for the 3.5%-fat-corrected milk, milk fat yield and milk fat % compared to the MC cows. The total income, cost of dry matter and income over feed costs per cow/d were higher in the control vs. SDG simulated dairy herds. The control herd had a higher income over feed costs than the SDG herd because the average milk yield per cow/d was higher even though the SDG herd had a lower total mixed ration cost and higher milk fat production.

## 1. Implications

Solvent-extracted dried distillers grains can be used to partially subsitute for canola meal without increasing the risk of milk fat depression. Substituting canola meal with solvent-extracted distillers grain with solubles resulted in a cheaper ration than that of the control, but, since the control ration had a higher milk yield, the control’s income over feed costs was greater than that of the solvent-extracted distillers grain with solubles.

## 2. Introduction

Due to the high unsaturated fatty acid content of dried distillers grains with solubles, their level of inclusion in total mixed rations and use as a protein source are limited. The high unsaturated fat content in dried distillers grain with solubles increases the risk of milk fat depression and causes negative effects on rumen digestion [1]. However, several extraction processes could be used to reduce these negative effects. The composition of dried distillers grains with solubles with 28% crude protein (CP) and 9% dry matter (DM) ether extract (EE) [2] can be altered by removing the bran and germ before fermentation, which produces high-protein dried distillers grains with 41% CP and 5.4% DM EE [3]. Reduced-fat dried distillers grains involve removing the corn oil from dried distillers grains with solubles using centrifugation and have 34% CP and 7.4% DM EE [4], or the fat can be removed using a solvent process to produce SDG with 34% CP and 2.7% DM EE [5]. 

Canola meal (CM) with 41% CP and 2.64% DM EE [6] is a common protein supplement that is high in lysine and rumen degradable protein (RDP) compared to dried distillers grains with solubles [7]. It has been associated with increased milk production compared to other protein supplements [8,9]. Since dried distillers grain products are associated with a high amount of rumen-undegradable protein (RUP), the combination of SDG and CM may improve milk and milk component yields compared to those obtained by including CM as the only protein supplement. Additionally, since the cost of protein supplements influences feeding decisions, knowing the impact of including SDG as a partial substitute for other protein sources, such as CM, could reduce total the feed cost, while maintaining milk production and components. Milk production results and economic analyses are critical for the decision-making process of nutritionists and farmers when balancing ingredients, feed costs, and cow performance. Since feed costs represent more than 60% of the total costs of milk production, the income over feed costs can be used as a proxy for profitability and is useful in this decision-making process. Partial budget analyses and risk analyses have been used by other researchers to evaluate the economic benefits of substituting ingredients in dairy diets [10,11]. Therefore, the hypothesis of this study is that the partial replacement of CM with SDG in the total mixed ration will improve milk production and be more profitable than including CM alone in the total mixed ration. The first objective of this study was to substitute 70% of CM in the cows’ diets in a commercial dairy herd with SDG and urea to make their diets isonitrogenous and evaluate the impact of this substitution on the milk yield and milk component production. The second objective was to use partial budget analyses based on the data from the cow study in objective 1 and historical feed prices to evaluate if it would be more profitable for a dairy farm to partially substitute canola meal with SDG.

## 3. Material and Methods

### 3.1. Experimental Design and Treatments for the Cow Study

A total of 1408 Holstein cows were randomly enrolled to one of four dietary treatment groups: (1) primiparous control (PC) with 13% CM and 0.21% urea; (2) primiparous SDG cows (PSDG) fed 4.2% CM, 8.78% SDG and 0.42% urea; (3) multiparous control cows (MC) with 12.8% CM and 0.21% urea; and (4) multiparous SDG cows (MSDG) with 4.13% CM, 8.63% SDG and 0.42% urea. Urea was added to the total mixed ration to make the total mixed ration isonitrogenous within parity treatments.

The experimental design was randomized by even/odd ear tag number for each block. Block was defined as treatment period and consisted of 4 pens with 1 pen per treatment parity. Block 1 data collection began at 3 wk, and then cows remained on their treatments for the following 6 wk. At the end of block 1, PC and PSDG total mixed rations were switched and MC and MSDG total mixed rations were switched, and pens were fed their respective total mixed rations for a 2-week acclimation period. Data collection for block 2 began after the second acclimation period and continued for the next 6 weeks for a total data collection time of 12 wk. Therefore, considering both blocks, there were 2 pen replicates for each treatment. 

### 3.2. Cow Management, Housing and Milking

The study was conducted on commercial dairy farm with 6000 milking cows in the San Joaquin Valley of California from November of 2018 to February of 2019. Lactating primiparous and multiparous cows were housed separately in 2 primiparous pens averaging 362 cows per pen and 2 multiparous pens averaging 353 cows per pen. The pen capacity was 380 cows with fans on every other pole approximately 12.2 m apart and soakers above all headlocks. Cows had ad libitum access to water and no access to outside loafing pens during the trial. 

### 3.3. Total Mixed Ration and Feed Analyses

Cows were fed twice daily starting at 05:50 for morning feedings and 12:30 for afternoon feedings. The total mixed rations were pushed up every 2 h, and refusals were collected and measured daily. The total mixed rations offered and refused were recorded daily using the EZFeed software (v. 12.2.2205.1 Amelicor, Provo, UT, USA), and daily pen head counts were recorded using Dairy Comp 305 (Valley Ag. Software, Tulare, CA, USA). Average daily DMI was estimated by calculating feed offered minus refusals divided by the number of cows in the pen. Feed samples of forages (alfalfa hay and corn silage), SDG, and CM were collected every 2 wk, and total mixed ration samples were collected 1/wk via the tub sampling method [12]. The SDG and CM samples were stored at 20 °C and sent out for analyses to Cumberland Valley Analytical Services Inc. (Cumberland Valley Analytical Services Inc., CVAS, Maugansville, MD, USA) and Rock River Laboratory West Inc. (Visalia, CA, USA) for RUP and fatty acid analyses. The weekly total mixed ration, forage and composite SDG and CM CVAS samples were analyzed for DM, fat (via EE), CP, ADF and mineral analyses (Ca, P, Mg, K, S, Na, Cl, Fe, Cu, Mn, and Zn) using inductively coupled plasma mass spectrophotometry [13] method 2003.05, 941.04,973.18, 985.01 for Ca, P, Mg, K. Na, Fe, Cu, Mn and Zn; method 923.01 for S; and method 915.01 for Cl, aNDF and aNDFom [14], sugar [15], total fatty acids and fatty acid profile [16], lignin [17], soluble protein (SP) [18] and starch [19]. Composite SDG and CM samples were also analyzed for total fatty acids, fatty acid profile [16] and evaluated for AA and RUP digestibility via Ross RUP method [20] and RUP [21]. Because SDG has a smaller particle size than CM, the SDG samples from Novita (Brookings, SD, USA) were sent to CVAS and evaluated for AA and RUP digestibility using a different multi-step modified Ross RUP method, where RUP was determined by material recovered by freeze drying. This method lowered the probability of residual SDG not being captured on the small-pore filter paper resulting in underestimating RUP.

### 3.4. Milk Production

Milking was carried out twice daily in an 80-cow rotary milk parlor equipped with fans and sprinklers. The first milking started at 10:00 and finished at 13:00, the second milking started at 22:00 and finished at 01:00. Daily milk yields were collected from Dairy Comp 305 (Valley Ag. Software, Tulare, CA, USA) and averaged weekly for each pen. Milk components (fat, protein, lactose, SCC, SNF and MUN) were analyzed weekly by Tulare County Dairy Herd Improvement Association from individual cow milk samples collected from the morning milking. Fat, protein and lactose were analyzed using mid-infrared spectroscopy (Bentley 2000 Infrared Milk Analyzer, Bentley Instruments, Chaska, MN, USA). Milk urea N were determined using chemical methods based on a modified Berthelot reaction (ChemSpec 150 Analyzer, Bentley Instruments). Somatic cell counts were analyzed with a flow cytometer laser (So-macount 500, Bentley Instruments). Energy-corrected milk was determined using the equation (0.327 × kg of milk) + (12.95 × kg of fat) + (7.2 × kg of protein), and 3.5% FCM was determined using the equation (0.432 × kg of milk) + (16.23 × kg of fat); both equations were derived from [22].

### 3.5. Body Condition Scoring

Body condition scores were estimated weekly and determined by the same two individuals, using a 1-to-5 scale [23].

### 3.6. Sample Size Determination and Exclusion Criteria

A sample size of 312 cows per pen block was determined assuming an 8% increase in FCM in multiparous cows in response to substitution of 70% of CM with SDG, a standard deviation of 19 kg/d of 3.4% FCM, an alpha of 0.05 and power of 0.80. 

To be included in the data analyses, a cow had to be present in the pen during the acclimation period followed by at least 2 sequential wk during the data collection period. Data were removed from cows that were ill, i.e., moved to the hospital pen, were lame, died or were culled.

### 3.7. Statistical Analysis for the Cow Study

Pen was the experimental unit of interest. All averages were presented as least squares means. Differences were defined at *p* ≤ 0.05. Normality of dependent variables was evaluated using the Shapiro–Wilk test statistic and normal probability plots. None of the dependent variables were non-normal distributions, therefore transformation of the variables was not necessary.

To establish that total mixed ration nutrient composition was consistent per treatment throughout 12-week data collection period of the study, the MIXED procedure of Statistical Analysis System (SAS; v.9.4, SAS Institute, 2019, Cary, NC, USA) was used to evaluate differences in nutrient composition of the total mixed ration within parity (Table 1). Independent variables were each nutrient, and dependent variables were treatment (PC vs. PSDG or MC vs. MSDG), block (1 or 2) and repeated measures by week (*n* = 12, 6 per block). 

To determine the impact of partially substituting CM with SDG and urea as protein sources on milk yield and components, the data of primiparous and multiparous cows were analyzed separately using the MIXED procedure of SAS. Dependent variables were weekly milk yield, energy-corrected milk (ECM), 3.5%-fat-corrected milk (FCM), fat yield, fat %, protein yield, protein %, solids-not-fat (SNF) yield, SNF %, somatic cell count (SCC), milk urea nitrogen (MUN), lactose yield, lactose %, dry matter intake (DMI), average daily gain (ADG) and body condition score (BCS) change. Independent variables were treatment, block, days in milk (DIM) and days with treatment, with repeated measures for cows nested within pens per week. Independent variables that were not significant (*p* > 0.05) were removed from the analyses by backwards elimination.

### 3.8. Economic Model Methods

A partial budget analysis was developed in Excel to compare income over feed costs (IOFC) per cow/d. The budget analyses were based on a theoretical herd of 1000 lactating Holstein cows composed of 35% and 75% primiparous and multiparous cows, respectively. Results from Table 2 were used as inputs for the economic analysis. In the analyses, cows were only fed PC, MC, PSDG or MSDG during the 100 d in the high-producing group based on data from the commercial cow study. Inputs and assumptions used in the simulations are described in Table 3.

### 3.9. Milk Production and Feed Intake for the Economic Model

Lactation curves were estimated using Wood’s incomplete gamma curve [24] based on peak milk yield, DIM at peak and persistency (% decline) from the commercial cow herd study. Curves resembled the observed data from the trial; PC and MC average milk production for high-milk-producing cows was 36.4 and 48.6 kg, respectively. The calculated peak yields were lower for PC vs. MC cows due to parity differences: 36.7 and 49.9 kg per cow/d, respectively. Days in milk at peak were assumed to be 104 and 58 d, and monthly decreases in milk production were 3% and 6.35%, resulting in 305 d milk yields of 10,469 and 12,816 kg, respectively, for primiparous and multiparous cows (Table 3). For PSDG and MSDG cows, peak yields estimated using Wood’s incomplete gamma curve were 35.6 and 48.5 kg per cow/d, DIM at peak was 104 and 58 d and monthly decreases in milk production were 3% and 6.35% for primiparous and multiparous cows, respectively. The resulting 305 d milk yield was 10,367 and 12,692 kg, and average milk production for the high-producing cows was 35.3 and 47.2 kg for PSDG and MSDG cows, respectively.

### 3.10. Component Calculations for Treatment Groups in the Economic Model

Due to differences in milk production and composition between PC and MC as well as the PSDG and MSDG high-producing cows (Table 2), changes in bulk tank milk components were estimated. The overall average bulk tank milk components during the treatment period were obtained from farm milk shipping data (Land O’ Lakes, 2019) of fat, protein and SNF (3.7, 3.2, and 8.9%, respectively; Table 3). To estimate PSDG and MSDG fresh- and late-lactation cows, fat, protein and SNF percentages as well as the number of primiparous and multiparous cows in the fresh-, high-, and late-lactation groups were estimated by dividing the number of days spent in each group by the total number of DIM. For example, cows in high-producing pens represented 100/305 = 33% of all lactating cows. The total milk produced during the trial period, using the average milk produced by primiparous and multiparous cows in the fresh-, high-, and late-lactation groups, was estimated using data from the trial (Table 2) and from Wood’s lactation curve. Fresh- and late-lactation average milk yields were 33 and 33.7 kg per primiparous cow/d and 48.5 and 37.9 kg per multiparous cow/d (Table 4). Then, the contribution of bulk tank milk composition, milk fat, protein and SNF (Table 3) from PC, MC, PSDG and MSDG high-producing cows were used to estimate total milk income. 

### 3.11. Economic Model Inputs and Sensitivity Analysis

Total mixed ration DM costs were USD 0.274 and USD 0.270 kg per cow/d for PC and MC cows, respectively, and USD 0.268 and USD 0.264 kg per cow/d for the PSDG and MSDG cows, respectively. Feed prices were fixed and based on July 2019 market values (personal communication, Gavilon Animal Nutrition, Omaha, NE, USA) for both CM and SDG. The GoalSeek tool (Excel 2016, MicroSoft Corporation, Redmond, WA, USA) was used to estimate the value of SDG compared to that of CM.

The model used Federal Market Milk Order 51 (Sacramento, CA, USA), November 2018–December 2019 [28], to vary prices to see if changes in butter fat, protein and other solids would influence the value of SDG relative to that of CM. Considering this was the only large commercial dairy feeding trial in which SDG was used, the economic analysis was only based on data from this study. To account for possible variations in the results observed in this trial, a sensitivity analysis was created using a spreadsheet add-in program (version 5.0 @Risk, Palisade Corp., Raleigh, NC, USA) with 5000 iterations. The inputs milk yield, fat, protein, SNF percentages and associated standard errors observed in the research trial (Table 2) were used to generate a normal distribution curve. Based on the normal distributions of milk production and a fixed cost of feed, the economic model estimated IOFC (Table 4).

## 4. Results and Discussion

### 4.1. Nutrient Content, Total Mixed Ration Analyses and Dry Matter Intake

There were no differences in composition of the total mixed ration between the treatments within each parity over the 12 weeks of the study (Table 1). 

The substitution of the CM with the SDG and additional urea did not have any effects on the DMI between the treatments (Table 2). These results agree with those of other studies [29,30] that showed no differences in the DMI when primiparous and multiparous cattle were fed 20–30% SDG total mixed ration, replacing SBM as a protein source. 

The canola meal had a greater percentage of CP, SP, ADF, starch and RDP (as a percentage of CP) and a lower percentage of NDF and RUP (as a percentage of CP) when compared to those of the SDG (Table 3). Due to the removal of the corn oil in the extraction process to produce the SDG, there were lower amounts of total fatty acids and rumen unsaturated-fatty-acid load (RUFAL) [31] in the SDG vs. CM. There was no difference in the fat content of the total mixed ration (Table 1), and the milk fat yield was not impacted by the treatment in the primiparous cows. 

### 4.2. Milk Yield

The milk yield was lower for both the primiparous and multiparous SDG cows than that of the C cows (Table 2). All other studies that have included SDG in total mixed rations did not analyze primiparous and multiparous cows separately and compared SDG to SBM and not CM containing total mixed rations. Research using similar levels of CP diets (17–18%) has shown that feeding up to 30% SDG in total mixed rations has no effect on the milk yield when compared to SBM in total mixed rations [29,30].

The decrease in SDG multiparous and primiparous cow milk yields compared to those of the C cows in this study may be due to decreased lactose synthesis. In the mammary gland, protein and glucose supply are responsible for lactose production due to the importance of α-lactalbumin as a cofactor for lactose synthesis. The SDG multiparous and primiparous cows had lower lactose yields and higher milk protein yields when compared to those of the C cows in this study. However, the multiparous SDG cows had higher amounts of MUN, implying that their N utilization was not as great as that of the multiparous C cows. Since lactose is also the primary osmol for milk yield, the decrease in the SDG lactose yield may have contributed to the lower milk yield in the SDG vs. C treatments. Because the SDG total mixed ration had an increased inclusion rate of urea, there may not have been enough fermentable carbohydrate in the SDG total mixed ration to compensate for the additional SP coming from the urea in the SDG total mixed raton. 

### 4.3. Milk Fat Yield and Percentage

The primiparous SDG cows had a lower milk yield and milk fat yield compared to those of the primiparous C cows. The milk fat percentage was relatively high in both groups of the primiparous cows; therefore, milk fat depression was unlikely. The decrease in the milk yield for the PSDG cows was balanced with the decrease in the fat yield, resulting in no difference in the milk fat percentage or FCM. 

The multiparous SDG cows had a greater milk fat yield and percentage compared to that of the multiparous C cows, leading to a higher FCM. The increase in fat percentage and FCM was partially due to a lower, but not different, milk yield. Other studies comparing SBM to SDG that included about 30% total mixed rations have concluded that SDGs do not affect milk fat yield and percentage [30].

### 4.4. Milk Protein Yield and Percentage

The primiparous and multiparous SDG cows had a lower protein yield and percentage compared to those of the C cows. Similar to the milk fat percentage, the decrease in milk protein percentage is partially due to the increased milk yield. Morris et al. [4] evaluated the effects of the continuous feeding of high inclusion rates of reduced-fat dried distiller grains (RFDDG; centrifugation method) using 36 Holstein cows (9 primiparous, 27 multiparous) with 17.5% CP diets and also found that the milk protein yield decreased for the cows fed RFDDG with 29% total mixed rations compared to that of the cows fed SBM as the main protein source (1.28 vs. 1.32 kg, for the milk protein yield, respectively). The milk protein decreases in this study were likely a result of the SDG total mixed rations not having enough fermentable carbohydrates to compensate for the additional SP coming from the increased inclusion rate of urea in the SDG total mixed rations. The amount of carbohydrates available in the microbial cell is crucial to the microbial synthesis of AA which can be used for microbial growth, microbial protein yield, milk and protein yield. When including SDG in lactating dairy cattle’s total mixed rations, future research should consider formulating total mixed rations to include higher amounts of fermentable carbohydrate, especially when additional SP is used. This will aid microbial protein production in maintaining or increasing the protein yield.

### 4.5. Economic Analysis

Since only high-producing cow data from the current study were available, the data for the fresh- and late-lactation cows’ milk production and components were assumed to have no carryover effects due to their previous diets or dairy farm conditions. While the total cost of DMI was greater in for the C total mixed rstions compared to that of the SDG total mixed rations (Table 4), the IOFC was still greater for the C simulated cows (USD 3.14 per cow/d) than that of the SDG cows (USD 3.10 per cow/d). 

### 4.6. Sensitivity Analysis

**Figure 1:**
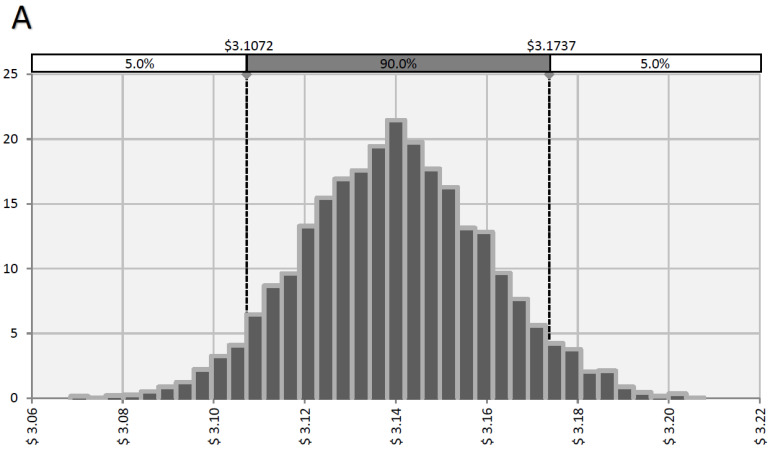

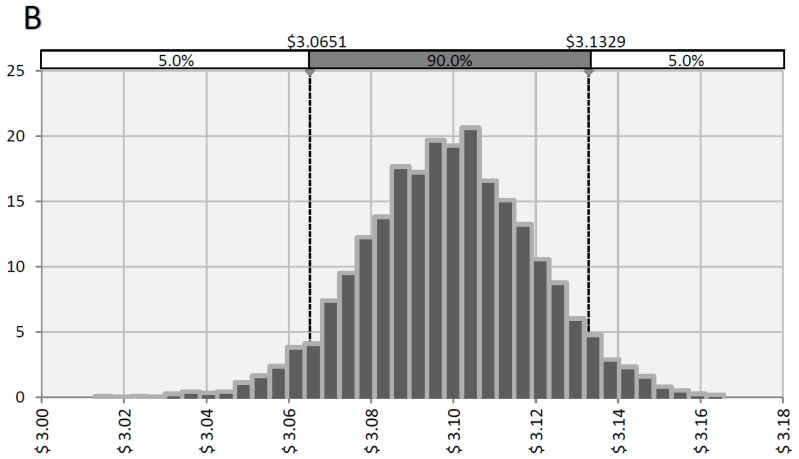
Sensitivity analysis showing the range of IOFC (USD per cow/d) for the control herd simulations (**A**) and solvent-extracted distillers grains with solubles (SDG) herd simulations (**B**). If no changes in model inputs are made, (**A**) 90% of the time, IOFC (USD per cow/d) varied from USD 3.11 to USD 3.17 for the control herd; and (**B**) 90% of the time, IOFC (USD per cow/d) varied from USD 3.07 to USD 3.13 for the SDG herd. Control herd simulations were based on milk and component yields from a 1000-cow herd fed the primiparous control (PC) and multiparous control (MC) diets from 14 DIM to 114 DIM. SDG herd simulations were based on milk and component yields from a 1000-cow herd fed the primiparous SDG and multiparous SDG diets from 14 DIM to 114 DIM.

To determine the importance of the inputs and assumptions (Table 4 and Table 5) relative to the outputs, a sensitivity analysis was performed. The inputs were held constant, and the milk and component prices were varied according to Table 6. Based on the changes in component prices from November 2018 to December 2019, 90% of the time the IOFC varied from USD 3.11 to USD 3.17 per cow/d when C total mixed rations were used (Figure 1A). When the SDG total mixed rations were fed, the IOFC varied from USD 3.07 to USD 3.13 per cow/d (Figure 1B). These results indicate that, based on this study, the C total mixed rations had a greater IOFC than that of the SDG total mixed rations. However, if the production is altered due to the treatments, total mixed rations, farm cow distribution or milk prices, the IOFC results would change.

**Figure 2:**
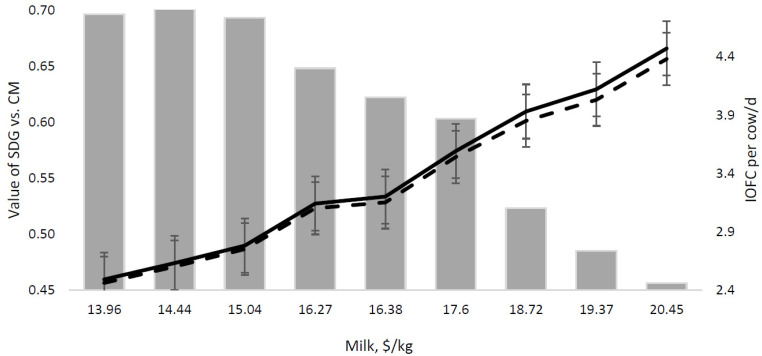
Simulated value of solvent-extracted distillers grains with solubles (SDG) vs. canola meal (CM) using [28] (Sacramento, CA, USA), November 2018–December 2019 [28] and income over feed cost (IOFC) per cow/d. Value of SDG vs. CM: SDG herd IOFC is ---- and control herd IOFC is 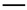.

Then, the fixed prices of the SDG and CM were assumed, and variations in the value of the SDG relative to CM were estimated (Table 6). As milk prices increased, the value of the SDG compared to that of the CM decreased and the IOFC increased in both the C and SDG simulated groups (Figure 2). The increase in the IOFC was not linear between the two groups. As milk prices increased, the differential in the IOFC increased, with the C group having a higher IOFC than that of the SDG simulated group.

Based on the model’s assumptions, the optimal point at which the SDG should be priced in relation to the CM was estimated. When considering differences in both the milk production and feed costs for the SDG and C simulated groups, it was estimated that the SDG would need to be valued at 65% of the value of the CM for the IOFC of SDG to break even to that of the C simulated group. The value of the SDG compared to CM was dependent on the variation in the total milk price, which was altered by the milk price components; thus, if an SDG total mixed ration increased the milk fat yield and the milk fat was consistently high, the SDG could be valued at a higher percentage of CM.

## 5. Conclusions

This economic model can provide an understanding of the value of a feed ingredient to a dairy given fluctuations in milk prices. As of July of 2019, SDG, CM and DDGS were priced at USD 226/ton, USD 284/ton and USD 195/ton, respectively (personal communication, Gavilon Animal Nutrition, Omaha, NE, USA). Using these values, the SDG was priced at 80% of the value of CM. Since the SDG is derived from the DDGS and requires additional processing costs to remove oil, this also suggests that the SDG should be valued at a price 14% higher than the DDGS. The value of these commodities is typically based on their nutrient profile, relative to protein composition and manufacturing costs, rather than their actual contribution to milk production. This economic model, however, can be used to evaluate SDGs based on their effects on milk production results, the milk yield and milk component price fluctuations compared to those of CM. When considering differences in both the milk production and feed costs for the SDGT and C simulated groups, it was estimated that SDGs would need to be valued at 65% to the value of CM for the IOFC of the SDGs to break even with that of the C simulated group.

## Figures and Tables

**Table 1:** Least squares mean ingredients and chemical composition of weekly treatment total mixed ration-fed cows during the study periods.

Item	PC ^1^	PSDG	SEM	*p*-Value	MC	MSDG	SEM	*p*-Value
Nutrient, % DM								
DM	51.4	51.6	1.14	0.92	53.1	53.3	1.91	0.98
CP	17.6	17.3	0.23	0.31	17.1	17.0	0.32	0.69
SP, % CP	27.5	30.4	0.83	0.07	27.7	30.1	0.99	0.07
ADICP	1.52	1.58	0.10	0.64	1.51	1.55	0.16	0.89
NDICP	1.79	1.86	0.27	0.84	1.85	2.01	0.29	0.68
ADF	17.9	17.3	0.52	0.40	17.9	17.6	0.49	0.58
NDF	26.9	27.0	0.64	0.92	26.6	27.0	0.60	0.54
Starch	25.9	26.4	0.82	0.62	26.7	26.7	0.63	0.96
NFC ^2^	43.2	43.7	0.89	0.60	45.0	44.6	0.67	0.60
Lignin	4.97	4.69	0.19	0.29	4.87	4.44	0.39	0.37
EE	5.64	5.88	0.25	0.44	5.51	5.47	0.15	0.79
Ash	8.45	7.91	0.25	0.09	7.84	7.88	0.24	0.86
Calcium	0.98	0.99	0.05	0.87	0.96	0.94	0.04	0.63
Phosphorus	0.52	0.52	0.02	1.00	0.49	0.49	0.02	0.94
Magnesium	0.47	0.46	0.02	0.66	0.45	0.44	0.01	0.37
Potassium	1.39	1.43	0.07	0.56	1.41	1.42	0.05	0.88
Sulfur	0.33	0.31	0.01	0.27	0.32	0.30	0.01	0.12
Sodium	0.69	0.66	0.04	0.56	0.64	0.66	0.03	0.43
Chloride	0.58	0.52	0.07	0.47	0.51	0.54	0.04	0.46
Iron, PPM	352	339	43.6	0.79	326	345	34.7	0.64
Manganese, PPM	99.3	93.9	3.77	0.29	101	91.9	5.65	0.25
Zinc, PPM	121	126	6.45	0.56	115	121	3.72	0.25
Copper, PPM	18.1	17.0	1.06	0.41	18.3	17.0	0.88	0.27
NE_l_, Mcal/kg	0.35	0.35	0.01	0.22	0.35	0.35	0.01	0.65
Ingredients, % total mixed ration								
Almond hulls	5.07	7.34			5.07	7.33		
Alfalfa hay	14.0	12.9			14.0	12.9		
Megalac ^2^	1.21	1.05			1.21	1.05		
Wet distillers grains	5.88	5.67			5.88	5.67		
Corn silage	18.2	16.3			18.2	16.3		
Rolled corn	24.2	25.9			24.2	25.9		
Wet citrus	1.84	1.73			1.84	1.73		
Pima cottonseed	6.25	6.14			6.24	6.13		
Canola meal	13.0	4.20			12.8	4.13		
SDG	0	8.78			0	8.63		
Corn gluten	5.46	5.37			5.46	5.37		
Control mineral ^3^	3.33	0			3.27	0		
SDG mineral ^4^	0	3.35			0	3.29		
Salt	0.42	0.41			0.42	0.41		
Molasses whey	1.20	1.17			1.19	1.17		

^1^ PC was primiparous control treatment; MC was multiparous control treatment; PSDG was primiparous solvent-extracted distillers grains with solubles treatment; and MSDG was multiparous solvent-extracted distillers grains with solubles treatment. Non fiber carbohydrate was calculated as NFC = 100 − (% NDF + % CP + % ether extract + % ash). EE was ether extract. SDG was solvent-extracted distillers grains with solubles. ^2^ Megalac (Arm and Hammer Nutrition Group, Princeton, NJ, USA). ^3^ Control mineral: 37.5% SQ 810 (Arm and Hammer Nutrition Group, Princeton, NJ, UAS), 30% calcium carbonate, 9.38% magnesium oxide 54%, 9.00% almond shell, 6.25% urea, 2.50% fat/oil, 2.50% dicalcium phosphate, 0.77% Zinpro Availa 4 (Zinpro, Eden Prairie, MN, USA), 0.55% zinc sulfate, 0.55% manganese sulfate, 0.33% Celmanax 3 g (Arm and Hammer Nutrition Group, Princeton, NJ, USA), 0.22% biotin 1%, 0.22% Sel Plex 2000 (Alltech, Inc. Nicholasville, KY, USA), 0.12% vitamin E, 0.05% copper sulfate, 0.05% selenium 1%, 0.03% trace salt with iodine (EDDI), 0.01% vitamin A and 0.01% vitamin D3. ^4^ SDG mineral: 37.5% SQ 810 (Arm and Hammer Nutrition Group, Princeton, NJ, USA), 30% calcium carbonate, 9.38% magnesium oxide 54%, 2.73% almond shell, 12.5% urea, 2.50% fat/oil, 2.50% dicalcium phosphate, 0.77% Zinpro Availa 4 (Zinpro, Eden Prairie, MN, USA), 0.55% zinc sulfate, 0.55% manganese sulfate, 0.33% Celmanax 3 g (Arm and Hammer Nutrition Group, Princeton, NJ, USA), 0.22% biotin 1%, 0.22% Sel Plex 2000 (Alltech, Inc. Nicholasville, KY, USA), 0.12% vitamin E, 0.05% copper sulfate, 0.05% selenium 1%, 0.03% trace salt with iodine (EDDI), 0.01% vitamin A and 0.01% vitamin D3.

**Table 2:** Production of Holstein cows fed a control or solvent-extracted distillers grains with solubles total mixed ration during the experimental period.

Item	PC ^1^	PSDG	SEM	*p*-Value	MC	MSDG	SEM	*p*-Value
Number of cows	356	340			357	355		
Days on treatment	42	42			42	42		
Milk, kg/d	36.4	35.3	0.17	0.01	48.6	47.2	0.21	0.01
ECM, kg/d	39.8	38.5	0.23	0.01	52.3	52.8	0.3	0.1
3.5% FCM, kg/d	40.7	39.5	0.46	0.34	53.3	54.4	0.35	0.01
Fat, kg/d	1.54	1.50	0.01	0.01	1.99	2.10	0.02	0.01
Fat, %	4.23	4.24	0.03	0.69	4.09	4.46	0.03	0.01
Protein, kg/d	1.04	1.00	0.01	0.01	1.39	1.33	0.01	0.01
Protein, %	2.86	2.84	0.01	0.01	2.89	2.87	0.01	0.01
SNF, kg/d	3.16	3.08	0.01	0.01	4.16	4.05	0.02	0.01
SNF, %	8.71	8.73	0.01	0.04	8.61	8.60	0.01	0.14
SCC,10^3^ cells/mL	140	149	12.8	0.51	188	192	13.4	0.78
MUN, mg/dL	11.6	11.4	0.08	0.01	9.38	9.74	0.09	0.01
Lactose, kg/d	1.78	1.74	0.01	0.01	2.31	2.26	0.01	0.01
Lactose, %	4.88	4.91	0.01	0.01	4.75	4.78	0.01	0.01
DMI, kg/d	23	23	0.05	0.33	30	30	0.05	0.46
ADG, kg/d	−0.15	0.25	0.33	0.23	0.19	0.01	0.15	0.22
BCS change	0.60	−0.02	0.54	0.25	0.64	−0.05	0.52	0.19

^1^ PC was primiparous control treatment; MC was multiparous control treatment; PSDG was primiparous solvent-extracted distillers grain with solubles treatment; and MSDG was multiparous solvent-extracted distillers grain with solubles treatment. Energy-corrected milk was calculated as ECM = [0.327 × milk yield (kg)] + [12.95 × fat (kg)] + [7.65 × protein (kg)] from [22]. The 3.5%-fat-corrected milk was calculated as FCM = [0.432 × milk yield (kg)] + [16.23 × fat (kg)] from [22].

**Table 3:** Chemical and fatty acid compositions of canola meal and solvent-extracted distillers grains with solubles.

Item	Canola Meal	SDG ^1^
Nutrient, % DM		
DM	89.3	87.3
CP	37.4	34.2
SP, % CP	35.7	19.9
ADF	16.2	15.8
NDF	25.4	35.6
Starch	5.10	3.70
EE	3.30	3.20
RUP, % CP	32.1	88.1
RUPD, % CP	66.2	74.9
RDP, % CP	67.9	11.9
Ash	6.70	6.00
Calcium	0.70	0.10
Phosphorus	1.00	1.00
Magnesium	0.50	0.40
Potassium	1.30	1.30
Sulfur	0.60	0.70
Sodium	0.10	0.20
Iron, PPM	142	98.0
Manganese, PPM	49.0	18.0
Zinc, PPM	69.0	61.0
Copper, PPM	61.0	4.00
Total fatty acids	3.80	3.18
RUFAL	3.35	2.59
Myristic (C14:0)	0.01	
Palmitic (C16:0)	0.26	0.51
Palmitoleic (C16:1)	0.05	0.01
Heptadecanoic (C17:0)	0.01	0.01
Heptadecanoic (C17:1)	0.01	
Stearic (C18:0)	0.05	0.05
Oleic (C18:1c9)	1.62	0.73
Oleic (C18:1w7)	0.52	0.03
Linoleic (C18:2w6)	0.95	1.72
Linoleic (C18:2w4)	0.01	
Linolenic (C18:3w3)	0.20	0.06
Arachidic (C20:0)	0.01	0.01
Eicosenoic (C20:1)	0.02	0.01
Behenic (C22:0)	0.01	
Lignoceric (C24:0)	0.01	0.01
Nervonic (C24:1)		0.02
Other	0.07	0.02

^1^ SDG was solvent-extracted distillers grains with solubles, EE was ether extract, RUPD was rumen-undegradable protein digestibility and RUFAL was rumen unsaturated-fatty-acid load.

**Table 4:** Economic model assumptions and inputs to determine the impact on milk production and profitability of substituting canola meal with solvent-extracted distillers grains with solubles.

Item	Input	Source
Number of lactating cows	1000	Assumption
Culling rate, %	35	Pinedo et al., 2010 [25]
Receiving total mixed ration, d	100	Assumption
Primiparous cows, %	35	Based on study data
Multiparous cows,%	65	Based on study data
Cows in fresh pen, d	21	Based on study data
Cows in high milking pen, d	100	Based on study data
Cows in late lactation pen, d	184	Based on study data
Calving interval, d	365	Touchberry et al., 1959 [26]
Dry period length, d	60	Coppock et al., 1974 [27]
PC 305 d milk yield, kg	10,469	Wood, 1967 [24]
MC 305 d milk yield, kg	12,816	Wood, 1967 [24]
PSDG 305 d milk yield, kg	10,367	Wood, 1967 [24]
MSDG 305 d milk yield, kg	12,692	Wood, 1967 [24]
Bulk tank fat, %	3.7	Based on study data
Bulk tank protein, %	3.2	Based on study data
Bulk tank SNF, %	8.9	Based on study data
Class III price, USD/kg	0.359	FMMO, 2019 ^2^ [28]
Skim milk price, USD/kg	0.159	FMMO, 2019 ^2^ [28]
Butterfat price, USD/kg	5.84	FMMO, 2019 ^2^ [28]
Protein price, USD/kg	4.10	FMMO, 2019 ^2^ [28]
SNF price, USD/kg	0.37	FMMO, 2019 ^2^ [28]
PC ^1^ ration cost, USD/kg per cow/d	0.274	Personal C ^3^
MC ^1^ ration cost, USD/kg per cow/d	0.270	Personal C ^3^
PSDG ^1^ ration cost, USD/kg per cow/d	0.268	Personal C ^3^
MSDG ^1^ ration cost, USD/kg per cow/d	0.264	Personal C ^3^
Primiparous DMI, kg per cow/d	23	Based on study data
Multiparous DMI, kg per cow/d	30	Based on study data

^1^ PC was primiparous control treatment; MC was multiparous control treatment; PSDG was primiparous solvent-extracted distillers grains with solubles treatment; and MSDG was multiparous solvent-extracted distillers grains with solubles treatment. ^2^ FMMO, 2019 is Federal milk market order 51, June 2019. ^3^ Personal C is personal communication in June 2019 with Gavilon Animal Nutrition, Omaha, NE, USA.

**Table 5:** Estimation of change in income due to feeding the primiparous control and multiparous control total mixed rations or the primiparous solvent-extracted distillers grain with solubles and multiparous solvent-extracted distillers grain with solubles total mixed rations from 14 DIM to 114 DIM based on the economic model in a 1000-cow dairy farm.

Item	Control ^1^ Herd	SDG ^2^ Herd
Fresh cows		
Number of primiparous cows	24	24
Number of multiparous cows	45	45
Primiparous milk yield, kg per cow/d	33	33
Multiparous milk yield, kg per cow/d	48.5	48.5
Total milk yield, kg	296,582	296,582
High-production milking cows		
Number of primiparous cows	115	115
Number of multiparous cows	213	213
Primiparous milk yield, kg per cow/d	36.4	35.3
Multiparous milk yield, kg per cow/d	48.6	47.2
Total milk yield, kg	1,453,443	1,410,984
Late lactation cows		
Number of primiparous cows	211	211
Number of multiparous cows	392	392
Primiparous milk yield, kg per cow/d	33.7	33.7
Multiparous milk yield, kg per cow/d	37.9	37.9
Total milk yield, kg	2,197,744	2,197,744
Components from high cows		
Fat, kg	60,051	62,018
Protein, kg	41,879	40,374
SNF, kg	125,507	121,921
Components of fresh and late lactation cows		
Fat, kg	85,891	85,891
Protein, kg	84,449	84,449
SNF, kg	225,844	225,844
Totals		
Number of primiparous cows	350	350
Number of multiparous cows	650	650
Average milk yield, kg per cow/d	44.3	43
Average fat, %	3.70	3.79
Average protein, %	3.20	3.20
Average SNF, %	8.90	8.90
Total milk yield, kg	3,947,769	3,905,310
Total fat, kg	145,942	147,909
Total protein, kg	126,329	124,823
Total SNF, kg	351,351	347,766
Economic Impact		
Total income, USD	558,602	549,325
Ration cost, DM basis, USD	244,616	239,423
Income over feed cost (IOFC), USD	313,985	309,901
IOFC, USD/d	3140	3099
IOFC, USD per cow/d	3.14	3.10

^1^ SDG is solvent-extracted distillers grain with solubles treatment. All diets were fed from 14 DIM to 114 DIM in the economic model simulations. ^2^ Control represents the IOFC from the control herd fed primiparous and multiparous control total mixed rations from post fresh (14 DIM) to the detection of pregnancy (114 DIM) based on the study’s response in milk yield and milk components in USD per cow/d. SDG is solvent-extracted distillers grains with solubles and represents the IOFC from the SDG herd fed total mixed rations from post fresh (14 DIM) to the detection of pregnancy (114 DIM) based on the study’s response in milk yield and milk components from the primiparous and multiparous solvent-extracted distillers grains with solubles treatments in USD per cow/d.

**Table 6:** Simulated value of solvent-extracted distillers grains with solubles relative to canola meal based on the economic model results and historical changes in the prices of milk fat, milk protein, milk solids and milk yield from [28].

FMMO Date ^1^	Fat, USD	Protein, USD	Solids, USD	Milk, USD	C IOFC, USD ^2^	SDG IOFC, USD	Value of SDG vs. CM
November 2018	2.54	1.34	0.27	14.44	2.63	2.6	70%
January 2019	2.50	1.19	0.29	13.96	2.49	2.46	70%
March 2019	2.55	1.63	0.22	15.04	2.78	2.75	69%
May 2019	2.57	2.12	0.18	16.38	3.2	3.15	62%
June 2019	2.65	2.00	0.17	16.27	3.14	3.1	65%
August 2019	2.66	2.45	0.17	17.6	3.59	3.54	60%
October 2019	2.40	3.17	0.14	18.72	3.93	3.85	52%
November 2019	2.32	3.91	0.11	20.45	4.47	4.38	46%
December 2019	2.20	3.65	0.13	19.37	4.12	4.03	49%

^1^ Federal Market Milk Order 51 date (California), November 2018–December 2019 [28]. ^2^ Control represents the income over feed costs (IOFC) from the control herd fed primiparous control (PC) and multiparous control (MC) total mixed rations from post fresh (14 DIM) to the detection of pregnancy (114 DIM) based on the study’s response in milk yield and milk components from the PC and MC treatments in USD per cow/d. SDG is solvent-extracted distillers grain with solubles and represents the income over feed costs (IOFC) from the primiparous SDG cows and multiparous SDG cows fed SDG total mixed rations from post fresh (14 DIM) to the detection of pregnancy (114 DIM) based on the study’s response in milk yield and milk components from the PSDG and MSDG treatments in USD per cow/d.

## Data Availability

The data were not deposited in an official repository but are available upon request.
